# Unexplained recurrent diffuse bleeding diathesis as a manifestation of nonaneurysmal intimomedial mucoid degeneration

**DOI:** 10.1016/j.rpth.2026.106978

**Published:** 2026-09-25

**Authors:** Cynthia Frima, Jan Jaap Spijkstra, Bernadette Schurink, Adriaan G. Holleboom

**Affiliations:** 1 Department of Intensive Care Medicine, Amsterdam University Medical Center, Amsterdam, the Netherlands; 2 Department of Pathology, Amsterdam University Medical Center, Amsterdam, the Netherlands; 3 Department of Vascular Medicine, Amsterdam University Medical Center, Amsterdam, the Netherlands

**Keywords:** aneurysm, arteries, hematoma, hemorrhage, hemorrhagic shock

## Abstract

**Background:**

Intimomedial mucoid degeneration (IMMD) is an exceptionally rare and potentially life-threatening vasculopathy characterized by mucoid accumulation within the intima and media, destruction of elastic fibers, and subsequent arterial wall weakening. Previously reported cases have been associated with aneurysmal disease, often accompanied by an increased risk of intraoperative hemorrhage.

**Key Clinical Question:**

Should IMMD be considered in patients with bleeding of unknown cause despite the absence of aneurysms?

**Clinical Approach:**

We describe a patient with an isolated, recurrent, and unexplained severe diffuse bleeding diathesis in the absence of any detectable aneurysm. He ultimately died of multiorgan failure following profound hemorrhagic shock due to retroperitoneal bleeding. Postmortem histopathology revealed features consistent with IMMD despite the lack of aneurysmal changes.

**Conclusion:**

This case highlights a broader clinical expression of IMMD than previously recognized by demonstrating that recurrent severe bleeding may occur in the absence of aneurysmal disease. It underscores the importance of further case reporting to improve future diagnosis and management.

## Essentials

• Intimomedial mucoid degeneration (IMMD) is a rare blood vessel disease linked to aneurysms.
• We report a patient with repeated, unexplained severe bleeding but no aneurysms.
• The patient died from internal bleeding, and examination after death showed signs typical of IMMD.
• IMMD can occur without aneurysms and may instead cause unexplained severe bleeding.

## 1 Introduction

Intimomedial mucoid degeneration (IMMD) is a rare and poorly understood vascular disorder characterized by the deposition of mucoid in the intima and media of blood vessel walls, accompanied by destruction of elastic fibers. These vascular changes typically lead to aneurysmal degeneration, and bleeding diathesis is commonly observed in the perioperative period [[1], [2], [3], [4], [5], [6], [7]]. First described by Decker et al. in 1997 [1], IMMD predominantly affects females and individuals of African descent, although cases across sexes and ethnicities have been reported [[3], [4], [5],^8^,^9^]. The abdominal aorta is most often involved, but both large aortic branches and smaller arteries have also been described [[4], [5], [6], [7], [8], [9], [10]].

In this report, we present the case of a Caucasian male patient who experienced recurrent, severe, and unexplained bleeding diathesis over several years. Ultimately, he succumbed to multiorgan failure caused by profound hemorrhagic shock due to retroperitoneal bleeding. Postmortem examination revealed histologic findings consistent with IMMD, although no aneurysms were observed. This case may indicate a broader clinical expression of IMMD than previously recognized.

## 2 Case Report

Since the age of 53, an otherwise healthy Caucasian male patient began to experience recurrent small hematomas following minor trauma. At age 58, he sustained his first severe hematoma after a bicycle accident. He was hospitalized and treated with blood transfusions due to significant hematomas in his quadriceps and gluteal muscles. Extensive hemostatic tests revealed no underlying cause of the bleeding (Supplementary Table), and computed tomography scan showed no evidence of aneurysms, arteriovenous malformations, or vascular occlusions.

Over the following months, he was readmitted multiple times due to hematomas from either minor trauma or spontaneous onset. Eventually, he developed a severe spontaneous retroperitoneal hematoma originating from his right iliopsoas muscle. Diagnostic and therapeutic decisions regarding hematomas and required interventions were guided by a recent review on bleeding of unknown cause and unclassified bleeding disorders [11]. During admission, he required multiple blood transfusions, underwent several angiographies, 3 embolizations, and 8 laparotomies due to rebleeding. Intraoperatively, diffuse bleeding was observed, and the abdomen was packed to achieve hemostasis. After prolonged treatments, including empirical use of tranexamic acid, high-dose activated eptacog alfa (Novoseven), and extended immobilization, he developed bilateral pulmonary embolisms and multiple thromboses in the jugular vein, subclavian vein, and superior vena cava. These were treated with prophylactic low-molecular-weight heparin and a temporary vena cava filter. Once his bleeding had stabilized and he could commence extensive rehabilitation, he was switched to a reduced dose of rivaroxaban.

Repeated wide-ranging hemostatic tests still did not provide an explanation, and antiphospholipid testing was negative. Vitamin C levels were normal, Ehlers-Danlos DNA testing was negative. Autoimmune serology was negative, and peripheral immunophenotyping showed no monoclonal B or T cells (Supplementary Table). Multiple biopsies from the soft tissue of the abdomen, skin, duodenum, ileum, and stomach failed to show any signs of infection, lymphoma, other malignancies, amyloidosis, vasculitis, or abnormalities in the vascular walls. However, as no bleeding vessels were excised intraoperatively, histopathologic examination was not performed on those vessels. Because of the persistently unexplained cause of bleeding and the inability to definitively exclude vasculitis or myositis, he underwent 2 courses of methylprednisolone therapy.

At age 61, he underwent a laparoscopic cholecystectomy for cholecystitis under pragmatic prophylaxis with tranexamic acid and desmopressin. Three days after surgery, he developed a postoperative hemorrhage originating from the cystic artery. The bleeding was successfully treated with embolization, and after stabilization, 6 L of old hematoma was removed laparoscopically. Examination of the resected cystic artery, including electron microscopy, showed no abnormalities.

In the years that followed, the patient experienced multiple hemorrhages in his legs and arms, either from mild trauma or spontaneously, again leading to several hospital admissions during which he received blood transfusions and procoagulant treatment. The patient described hearing a snapping sound in the affected limbs multiple times, immediately after which a hematoma would occur. He had chronically reduced mobility and an inability to sit due to these large hematomas, and he required skin transplantation due to necrosis, with very slow wound healing. Careful drainage under prophylaxis and attempts at sclerosing old, persistent hematomas to improve rehabilitation and mobility were unsuccessful, and surgical evacuation was deemed too risky. Despite consultations with 2 other tertiary centers, no definitive diagnosis was established. Whole-exome sequencing, added to the prior diagnostic workup, revealed no explanatory mutations.

At age 65, he was once more admitted, now for a hemorrhage in his left hamstring and quadriceps muscles, which was treated with tranexamic acid, factor VIII/von Willebrand factor (Haemate P), and activated eptacog alfa (Novoseven). A few days later, he developed deep hemorrhagic shock from a bleeding in his left iliopsoas muscle, originating from several lumbar arteries. In addition, computed tomography scan revealed bilateral segmental pulmonary embolisms. Several lumbar arteries were embolized, and a new vena cava filter was placed. He received massive transfusions and was transferred to the critical care unit. He developed progressive multiorgan failure and, despite aggressive support with high-dose vasoactive medications and continuous veno-venous hemodialysis, he passed away 2 days later.

Postmortem examination revealed histologic findings consistent with the diagnosis of IMMD. Examined vessels (common, external, and internal iliac arteries; superficial epigastric artery; iliolumbar artery (Figure); coronary arteries; internal thoracic artery) showed increased mucoid pools in the intima and media (Alcian blue stain). An inhomogeneous loss of smooth muscle cells was also observed, confirmed by smooth muscle actin and Elastica van Gieson stains, which additionally demonstrated irregular fibrosis. Large arteries, including the iliac artery, showed similar vascular changes without significant atherosclerosis to account for the findings. Similar findings were also observed in the smaller vessels and coronary arteries. Importantly, no signs of inflammation were seen. Aside from the pulmonary embolisms, no arterial thrombi were detected. One vein, the popliteal vein, showed an organized thrombus. No aneurysms or arteriovenous malformations were identified in any of the examined vessels, in line with earlier imaging.

**Figure:**
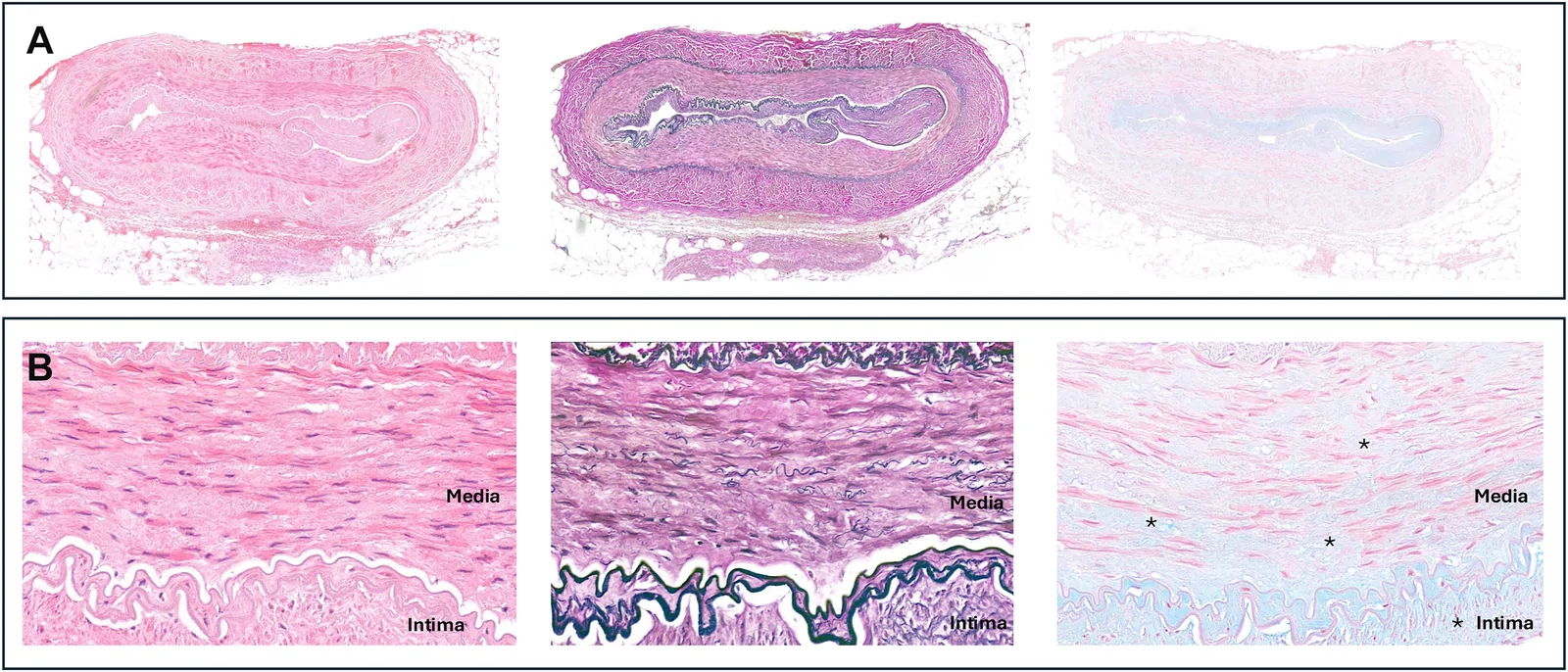
(A) Iliolumbar artery under low magnification, 50×. Left: hematoxylin and eosin (HE) stain. Middle: Elastica von Gieson (EvG) stain. Right: Alcian blue (A-blue) stain. Low-magnification view of the affected iliolumbar artery showing abnormalities of the arterial media, with loss of smooth muscle cells (HE), loss of elastin fibers (EvG), and increased deposition of mucoid pools in the media (A-blue). (B) Iliolumbar artery under high-power view, 400×. Left: HE stain. Middle: EvG stain. Right: A-blue stain. High-magnification view of the medial alterations in the affected iliolumbar artery with loss of smooth muscle cells (HE), loss of elastin fibers (EvG), and increased deposition of mucoid pools in the media (A-blue, asterisks). Although the media was mainly affected, A-blue staining was also seen in the intima.

## 3 Discussion

IMMD is a rare condition whose underlying etiology remains unclear. To date, approximately 60 IMMD cases have been reported. Additionally, older studies have described cases showing similar histopathologic findings [[12], [13], [14]]. Histologically, IMMD is characterized by progressive mucin deposition within the inner 2 layers of the vessel wall, accompanied by loss of smooth muscle cells and degradation of elastic fibers in the affected arteries [[1], [2], [3],[8], [9], [10]]. This structural deterioration weakens the arterial wall, predisposing to aneurysm formation and increasing vulnerability to hemorrhage, particularly after trauma or during surgical intervention. A notable histopathologic hallmark—also observed in our patient—is the absence of local atherosclerosis or an inflammatory response.

Typically, patients present with arterial aneurysms and have a substantially elevated risk of an intraoperative bleeding diathesis distinct from diffuse intravascular coagulation. Our patient, however, presented solely with an unexplained bleeding diathesis and exhibited no aneurysms—neither during life nor on postmortem examination. Despite years of evaluation, including extensive hemostatic and connective tissue studies, no underlying disorder was identified. Prior reported cases similarly describe severe perioperative bleeding despite normal preoperative coagulation profiles, with vessel fragility often exacerbating hemorrhage during surgical manipulation [2,3,6]. In some cases, this has led to profound hemorrhagic shock and even death [2,3]. Meticulous preoperative planning and careful surgical technique are therefore essential. One prevailing hypothesis is that a localized fibrinolytic process occurs within the affected vessels, which could also explain the marked absence of local atherosclerosis [2].

Our patient first developed symptoms at the age of 53 years, which closely corresponds to the mean age of 52 years reported in each of the 2 largest case series [1,2] and across the published adult case reports. However, cases have been described across a broad age range and even in a young infant [15].

Although most case reports describe a single arterial aneurysm, some report multiple aneurysms throughout the body. For example, Katz et al. [9] reported a patient with aneurysms in the superior gluteal, ophthalmic, superficial temporal, and internal carotid arteries, as well as both lower extremities. Such findings support the notion that IMMD represents a systemic disease, as seen in our patient.

To our knowledge, this is the second reported case of nonaneurysmal IMMD. The first, described by Tzavellas et al. [4], involved a patient with a self-contained rupture of the proximal subclavian artery after using a power leaf blower. He underwent bypass surgery, and histopathology demonstrated IMMD. However, unlike our patient, he showed no bleeding tendencies beyond the arterial rupture, suggesting that our case may represent a distinct clinical presentation of IMMD.

Our patient developed pulmonary emboli and venous thromboses. Although IMMD is not typically associated with thrombotic events, Cooper et al. [10] reported a case involving a superimposed occlusive thrombosis of the subclavian artery. In our patient, the thrombotic events occurred during hospitalization and were likely multifactorial in origin. Prolonged immobilization and the empirical use of multiple hemostatic agents, including activated factor VII, may have contributed to their development. However, given the life-threatening hemorrhage and limited therapeutic options, these interventions were considered necessary despite the associated thrombotic risk.

The fatal iliopsoas hemorrhage originated from several iliolumbar arteries, which demonstrated histopathologic changes consistent with IMMD during postmortem examination. Similar changes were also identified in smaller arteries, including the superficial epigastric artery. However, IMMD could not be histologically confirmed in the small intramuscular arteries, as these vessels were too small to be reliably identified during dissection. Nevertheless, the recurrent hematomas of varying size across multiple muscle groups suggest that these vessels may also have been affected, with the lack of histologic confirmation likely reflecting technical limitations rather than the absence of disease.

In retrospect, a biopsy of a larger affected artery from a (previous) bleeding site might have established the diagnosis of IMMD during life. However, obtaining such a biopsy was considered too high-risk in a patient with recurrent severe and, at times, life-threatening hemorrhage. None of the bleeding vessels were surgically excised and were therefore unavailable for histopathologic examination. A segment of the cystic artery, obtained after cholecystectomy, was examined both histologically and by electron microscopy but showed no abnormalities. In future cases, the potential diagnostic value of vascular biopsy should be carefully weighed against the considerable risk of hemorrhagic complications.

In conclusion, IMMD represents an extremely rare and potentially life-threatening condition with an unclear etiology. As IMMD is so rare and shows few distinctive clinical signs, diagnosis is difficult and often delayed, predominantly relying on histopathologic evaluation. Typically, patients present with aneurysmal disease. Management can be difficult as well, requiring avoidance of trauma and medical procedures as much as possible, along with prophylaxis and multidisciplinary care. To our knowledge, this is the first documented case presenting solely with an unexplained recurrent diffuse bleeding diathesis. This observation expands the known clinical spectrum of IMMD and highlights the crucial need for precise documentation of new cases, thereby advancing our understanding of the disease and improving future patient management.
