# Supplementary material for: Unexplained recurrent diffuse bleeding diathesis as a manifestation of nonaneurysmal intimomedial mucoid degeneration
Source: Res Pract Thromb Haemost. 2026 Sep 25;10(7):106978. doi: 10.1016/j.rpth.2026.106978 (PMC13691139; doi:10.1016/j.rpth.2026.106978)
Supplement: Supplementary file 1 [file mmc1.pdf]

Supplementary Table - Laboratory investigations

Test	Result[1]	Reference range
**Routine hematology and coagulation**		
Hemoglobin	6,1 mmol/L (9,8 g/dL), with substantial variation during follow-up.	8,5 – 10,5 mmol/L
Platelet count	187 x 10^9/L	150 – 400 x 10^9/L
Activated partial thromboplastin time (aPTT)	24,6 seconds	22 – 29 seconds
Prothrombin time (PT)	10,4 seconds	9,7 – 11,9 seconds
International normalized ratio (INR)	0,99	
Thrombin time (TT)	16,0 seconds	0 – 21 seconds
Fibrinogen	2,6 g/L	1,5 – 4,0 g/L
D-dimer	0,5 mg/L	0 – 0,5 mg/L
**Coagulation factor assays**		
Factor II activity	114 %	80 – 140 %
Factor V activity	132 %	80 – 140 %
Factor VII activity	111 %	80 – 140 %
Factor VIII activity	194 %	50 – 150 %
Factor IX activity	161 %	50 – 150 %
Factor X activity	121 %	80 – 140 %
Factor XI activity	105 %	50 – 150 %
Factor XII activity	101 %	50 – 150 %
Factor XIII activity	88 %; no evidence of a factor XIII inhibitor.	60 – 140 %
**Von Willebrand factor and primary hemostasis**		
Von Willebrand factor (VWF) activity	198 %	50 – 150 %
Von Willebrand factor (VWF) antigen	209 %	50 – 150 %
Von Willebrand factor (VWF) collagen binding assay	301%	51 – 181 %
Duke bleeding time	8 minutes (according to the laboratory report, this value was considered consistent with the low hemoglobin level of 5,0 mmol/L at the time of testing)	0 – 6 minutes
**Platelet function studies**		
Platelet function analyzer (collagen-adenosine diphosphate) (PFA ADP)	86 seconds	71 – 118 seconds
Platelet function analyzer (collagen-epinephrine) (PFA EPI)	62 seconds	85 – 165 seconds
Platelet aggregation tests (ADP, arachidonic acid, collagen, ristocetin)	No abnormalities	
Adenosine diphosphate (ADP)	3,0 μmol/10^{11} platelets	1,7 – 4,0 μmol/10^{11} platelets
Adenosine triphosphate (ATP)	4,3 μmol/10^{11} platelets	2,8 – 5,7 μmol/10^{11} platelets
ATP/ADP ratio	1,42	
**Fibrinolytic system**		
Plasminogen activity	85 %	80 – 140 %
Tissue plasminogen activator (t-PA) activity after venous occlusion	3,5 IU/mL	0,7 – 7,4 IU/mL
Plasminogen activator inhibitor-1 (PAI-1) antigen	23,5 ng/mL	3,4 – 39,0 ng/mL
Plasminogen activator inhibitor-1 (PAI-1) activity	12-16 U/mL. No evidence of a PAI-1 inhibitor.	2,0 – 7,0 U/mL
Alpha-2-antiplasmin activity	117 %	80 – 140 %
Euglobulin clot lysis time (ECLT) before venous occlusion	>300 minutes	>116 minutes
Euglobulin clot lysis time (ECLT) after venous occlusion	91 minutes	18 - 310 minutes

Euglobulin clot lysis time (ECLT) ratio	>3,3	1,2 – 5,7
**Natural anticoagulants**		
Antithrombin	76 %	80 – 140 %
Protein C activity	124 %	70 – 120 %
Protein S free antigen	55 %	81 – 147 %
**Antiphospholipid antibody testing**		
Lupus anticoagulant	Negative	
Anticardiolipin immunoglobulin G	Negative (<10U/mL)	
Anticardiolipin immunoglobulin M	Negative (<10U/mL)	
Anti-beta2-glycoprotein I immunoglobulin G	Negative (<7 U/mL)	
Anti-beta2-glycoprotein I immunoglobulin M	Negative (<7 U/mL)	
**Autoimmune and vasculitis serology**		
Anti-nuclear antibody (ANA)	Negative	
Antineutrophil cytoplasmic antibodies (ANCA), anti-myeloperoxidase (A-MPO) and anti-proteinase 3 (A-PR3)	Negative	
Anti-glomerular basement membrane (A-GBM)	Negative	
Anti-double-stranded DNA (A-dsDNA)	Negative	
Anti-centromere protein B (A-CENP-B)	Negative	
Anti-Jo-1 (A-JO-1)	Negative	
Anti-scleroderma-70 (A-SCL-70)	Negative	
Anti-Sjögren's syndrome related antigen A (A-SSA 60+52)	Negative	
Anti-Sjögren's syndrome related antigen B (A-SSB)	Negative	
Anti-mitochondrial antibody subtype M2 (A-AMA-M2)	Negative	
Anti-proliferating cell nuclear antigen (A-PCNA)	Negative	
Anti-polymyositis/scleroderma (A-PMSCL)	Negative	
Anti-ribosomal P protein (A-RIB-P)	Negative	
Complement component 1q	65 IE/mL	81 – 128 IE/mL
Complement C3	1,2 g/L	0,9 – 1,8 g/L
Complement C4	188 mg/L	150 – 400 mg/L
Anti-Smith (A-Sm)	Negative	
Immunoglobulin M rheumatoid factor (IgM-RF)	Negative	
Anti-immunoglobulin A	Negative	
Myositis immunoblot (Mi-2alpha, Mi-2beta, TIF1gamma, MDA5, NXP2, SAE1, Ku, PM-Scl (100kD), PM-Scl (75 kD), Jo-1, SRP, PL-7, PL-12, EJ, OJ, Ro52)	Negative	
**Direct antiglobulin testing**		
Direct antiglobulin test (DAT)	Weakly positive	
Anti-immunoglobulin G	Weakly positive, titer < 1:10	
Anti-immunoglobulin A	Negative	
Anti-immunoglobulin M	Negative	
C3c	Negative	
C3d	Negative	
**Additional hematologic investigations**		
Monoclonal gammopathy testing: Serum protein electrophoresis,	No evidence of monoclonal gammopathy	

monoclonal protein typing, free kappa, free lambda, free kappa-free lambda ratio		
Peripheral blood immunophenotyping	No clonal B cells and no abnormal T cells	
Immunoglobulin A	1,36 g/L	0,7 – 4,0 g/L
Immunoglobulin G	4,6 g/L	7,0 – 16,0 g/L
Immunoglobulin M	0,64 g/L	0,4 – 2,30 g/L
**Miscellaneous**		
Haptoglobin	0,77 g/L	0,3 – 2 g/L
Bilirubin (total)	16 µmol/L	4 – 24 µmol/L
Vitamin C	7,7 mg/L	
Creatinine	95 µmol/L	64 – 104 µmol/L
Human immunodeficiency virus (HIV)	Negative	
Treponema pallidum	Negative	
**Exome sequencing**		
Exome sequencing with analysis restricted to 159 genes; genes listed below[2]	No pathogenic or likely pathogenic variants were identified	

Footnotes

1) Most investigations were repeated during follow-up and subsequent second and third opinions, without identifying an underlying cause of the bleeding tendency.

2) Tested genes: A2M, ABCG5, ABCG8, ACBD5, ACTB, ACTN1, ACVRL1, ADAMTS13, ANKRD26, ANO6, AP3B1, AP3D1, APOLD1, ARPC1B, BLOC1S3, BLOC1S5, BLOC1S6, BRAF, C3, CALR, CBL, CD36, CD46, CDC42, CFB, CFH, CFI, CHST14, COL1A1, COL3A1, COL5A1, COL5A2, CTLA4, CYCS, DGKE, DIAPH1, DNASE1, DTNBP1, ENG, EPHB2, ERG, ETV6, F10, F11, F12, F13A1, F13B, F2, F2RL3, F5, F7, F8, F9, FBN1, FERMT3, FGA, FGB, FGG, FLI1, FLNA, FYB1, GALE, GATA1, GATA2, GBA1, GDF2, GFI1B, GGCX, GNE, GP1BA, GP1BB, GP6, GP9, HABP2, HOXA11, HPS1, HPS3, HPS4, HPS5, HPS6, HRG, IKZF5, ITGA2, ITGA2B, ITGB3, JAK2, KDSR, KLKB1, KNG1, KRAS, LMAN1, LYST, LZTR1, MASTL, MCFD2, MECOM, MLPH, MPIG6B, MPL, MTHFR, MYH9, MYO5A, NBEA, NBEAL2, NFE2, NRAS, OCRL, ORAI1, P2RY12, PIGA, PLA2G4A, PLA2G7, PLAT, PLAU, PLG, PRKACG, PROC, PROS1, PROZ, PTGS1, PTPN11, PTPN22, PTPRJ, RAB27A, RAF1, RASGRP2, RBM8A, RIT1, RNU4ATAC, RUNX1, SERPINC1, SERPIND1, SERPINE1, SERPINF2, SH2B3, SLFN14, SMAD4, SOS1, SOS2, SRC, STAB2, STIM1, STXBP2, TALDO1, TBX1, TBXA2R, TBXAS1, THBD, THPO, TNXB, TPM4, TREX1, TUBB1, VIPAS39, VKORC1, VPS33B, VWF, WAS, WIPF.
